# Reduced post-operative DPP4 activity associated with worse patient outcome after cardiac surgery

**DOI:** 10.1038/s41598-018-30235-w

**Published:** 2018-08-07

**Authors:** Heidi Noels, Wendy Theelen, Marieke Sternkopf, Vera Jankowski, Julia Moellmann, Sandra Kraemer, Michael Lehrke, Nikolaus Marx, Lukas Martin, Gernot Marx, Joachim Jankowski, Andreas Goetzenich, Christian Stoppe

**Affiliations:** 1 Institute for Molecular Cardiovascular Research (IMCAR), University Hospital Aachen, RWTH Aachen University, Aachen, Germany; 2 Department of Internal Medicine I-Cardiology, University Hospital Aachen, Aachen, Germany; 3 Department of Thoracic, Cardiac and Vascular Surgery, University Hospital Aachen, RWTH Aachen University, Aachen, Germany; 4 Department of Intensive Care Medicine, University Hospital, RWTH Aachen University, Aachen, Germany; 5 Cardiovascular Research Institute Maastricht (CARIM), Maastricht University, Maastricht, The Netherlands

## Abstract

Cardiac surgery with cardiopulmonary bypass (CPB) triggers myocardial ischemia/reperfusion injury contributing to organ dysfunction. Preclinical studies revealed that dipeptidyl peptidase (DPP4) inhibition is protective during myocardial infarction. Here, we assessed for the first time the relation of peri-operative DPP4-activity in serum of 46 patients undergoing cardiac surgery with patients’ post-operative organ dysfunction during intensive care unit (ICU) stay. Whereas a prior myocardial infarction significantly reduced pre-operative DDP4-activity, patients with preserved left ventricular function showed an intra-operative decrease of DPP4-activity. The latter correlated with aortic cross clamping time, indicative for the duration of surgery-induced myocardial ischemia. As underlying mechanism, mass-spectrometry revealed increased DPP4 oxidation by cardiac surgery, with DPP4 oxidation reducing DPP4-activity *in vitro*. Further, post-operative DPP4-activity was negatively correlated with the extent of post-operative organ injury as measured by SAPS II and SOFA scoring, circulating levels of creatinine and lactate, as well as patients’ stay on the ICU. In conclusion, cardiac surgery reduces DPP4-activity through oxidation, with low post-operative DPP4-activity being associated with organ dysfunction and worse outcome of patients during the post-operative ICU stay. This likely reflects the severity of myocardial ischemia/reperfusion injury and may suggest potential beneficial effects of anti-oxidative treatments during cardiac surgery.

## Introduction

Worldwide cardiovascular disease (CVD) is considered a major cause of morbidity and mortality, being responsible for 45% of non-communicable diseases^1^. Of estimated 17.7 million deaths caused by CVD in 2015, around 42% were due to coronary heart disease^2^. Patients increasingly present with comorbidities such as overweight, hypertension and type 2 diabetes mellitus (T2DM), which have been identified as important risk factors for CVD^3^. In particular the latter is known to be associated with an elevated risk of cardiovascular events, poor prognosis and increased mortality, with CVD responsible for at least 50% of deaths of diabetic patients^4^.

A significant number of these patients require cardiac surgery for the management of their heart disease^5^. However, patients undergoing cardiac surgery with cardiopulmonary bypass (CPB) are exposed to myocardial ischemia/reperfusion (I/R) injury after the termination of cardioplegic-induced myocardial arrest and reperfusion^6^. This induces perioperative inflammation and acute organ dysfunction^7,8^ and also affects long-term clinical outcome after surgery^8,9^. Improvements in surgical techniques reduced in-hospital patient mortality after cardiac surgery to ~2.6% in 2015^10^. Nonetheless, the high incidence of coronary heart disease and the growing prevalence of comorbidities increasing cardiovascular risk in an ageing population emphasize the need to study patients outcomes after cardiac surgery and the associated molecular mechanisms in more detail, as currently no effective therapy is available to tackle cardiac surgery-associated myocardial ischemia reperfusion injury^8^.

The dipeptidyl peptidase 4 (DPP4) is expressed in many organs and cell types, and exists in a membrane-bound form as well as in a soluble form in blood^11^. DPP4 cleaves N-terminal dipeptides from proteins with proline or alanine at the penultimate position, such as from glucagon-like peptide (GLP) -1 and CXCL12, thereby modulating their biological function^11,12^. Since GLP-1 plays a major role in glucose metabolism, DPP4 inhibitors are used for the treatment of T2DM with the aim to reduce the rate of GLP-1 inactivation and thereby reduce blood glucose levels^13^. Furthermore, DPP4 inhibition or genetic deficiency in animal models has been shown to exhibit anti-inflammatory and cardioprotective effects both through glucose-dependent as well as –independent mechanisms^12–15^, for example in the context of myocardial ischemia^16–19^. Furthermore, DPP4 inhibition could reverse left ventricular dysfunction and improve survival in animal models of chronic heart failure^20,21^. Accordingly, these studies suggest that high DPP4 activity levels negatively affect cardiovascular health. However, large-scale clinical trials examining the cardiovascular-protective effect of long-term DPP4 inhibitor treatment could not detect a reduction of cardiovascular events in patients with diabetes^22–27^, and some studies even surprisingly reported an increased hospitalization rate for heart failure^23,24,28^. This indicates that, despite attractive clinical features observed in animal models, the relation of DPP4 activity to clinical outcome requires further investigation in clinical practice.

In contrast to the wealth of animal studies focusing on DPP4 in inflammation and myocardial infarction, no data are available on the kinetics and clinical significance of DPP4 activity in patients undergoing cardiac surgery with CPB. Before advancing to a large-scale analysis to evaluate the effect of DPP4 inhibitors in this population, we aimed to provide first evidence about the peri-operative DDP4 activity and resulting clinical significance in patients undergoing cardiac surgery.

## Results

### Enrolled patients and baseline characteristics

**Figure 1:**
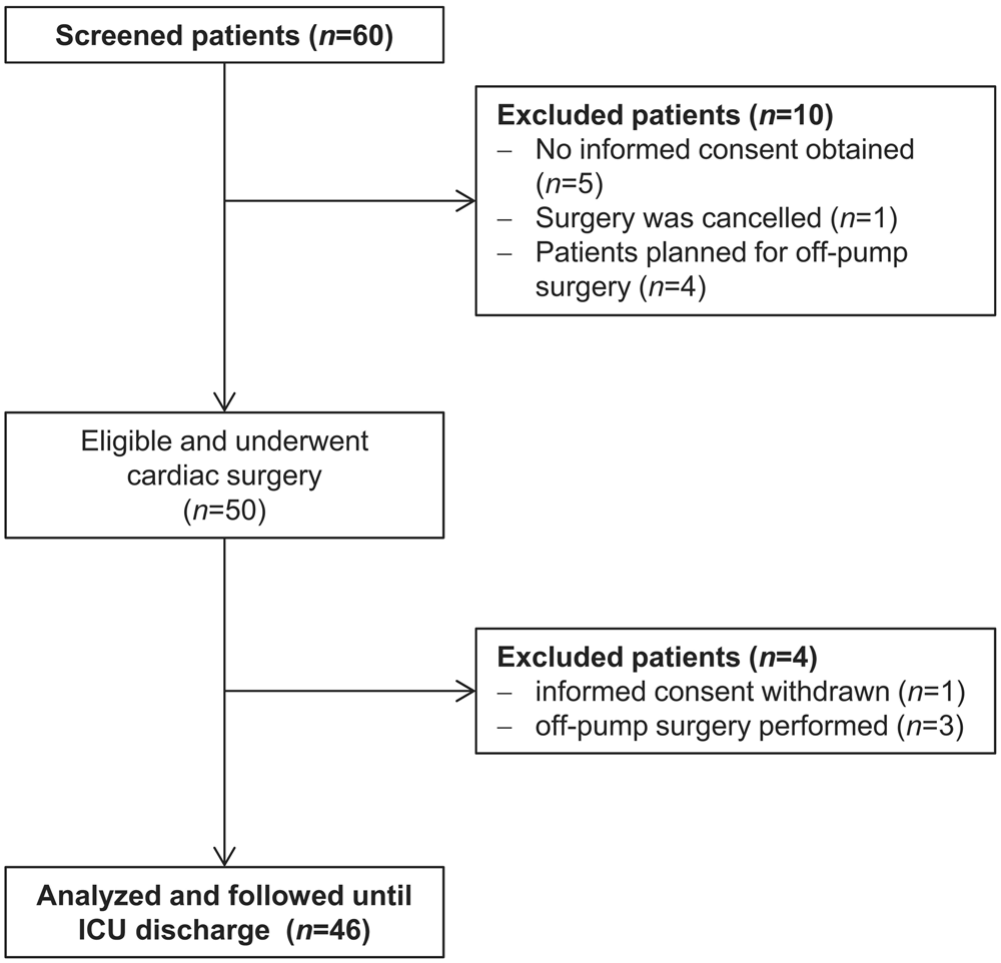
Flowchart of the present study. From initially 60 screened patients, 46 patients were enrolled.

From 60 screened patients, 50 patients scheduled for elective on-pump cardiac surgery were considered as eligible. Four patients were excluded from the following analysis because informed consent was withdrawn or the surgeon decided to perform surgery as a beating heart procedure (Fig. 1). Baseline characteristics of the patients are shown in Table 1. Sixty-five percent of patients were male and the median EuroSCORE for preoperative risk stratification was 5 (range 2–10). The included patients reflect a representative cohort of patients scheduled for elective cardiac surgery^29^. The type of surgery and intraoperative data (time of surgery, aortic cross clamp time) are depicted in Table 1. Of note, none of the patients was treated with DPP4 inhibitors.

**Table 1:** Baseline characteristics and data on surgery.

		All patients (n = 46)
Biometric/demographic data		
Age	years	69 ± 9
Sex, male	n (%)	30 (65)
Height	cm	170 ± 9
Weight	kg	80 ± 17
BMI	kg	27 ± 5
EuroSCORE I	median (range)	5 (2–10))
Medications		
Beta-blocker	n (%)	33 (72)
ACE inhibitor	n (%)	23 (49)
ASS	n (%)	36 (78)
Calcium antagonists	n (%)	10 (22)
Sartans	n (%)	7 (15)
DPP4 inhibitors	n (%)	0 (0)
Prior or preexisting disease		
Arterial hypertension	n (%)	27 (59)
Diabetes	n (%)	17 (37)
Smokers	n (%)	13 (28)
COPD or other obstructive lung diseases	n (%)	7 (15)
Pulmonary hypertension	n (%)	8 (17)
Chronic kidney disease	n (%)	7 (15)
Liver disease	n (%)	2 (4)
Unstable angina	n (%)	1 (2)
Acute endocarditis	n (%)	1 (2)
Recent myocardial infarction (within 90 days)	n (%)	17 (37)
LVEF >50%	n (%)	33 (72)
LVEF 30–50%	n (%)	8 (17)
LVEF <30%	n (%)	5 (11)
Type of surgery		
Isolated CABG	n (%)	29 (63)
Isolated valve surgery	n (%)	5 (11)
Others	n (%)	12 (26)
Intraoperative data		
Duration of surgery	min	265 ± 117
Aortic cross clamp time (myocardial ischemia time)	min	82 ± 51

Data are presented as median (range) (not normally distributed data), as mean ± SD (normally distributed data) or as absolute numbers (n) and respective percentage (%) of the whole. *ACE* = *angiotensin-converting-enzyme; ASS* = *Aspirin; BMI* = *body mass index; CABG* = *coronary artery bypass graft*; *COPD* = *chronic obstructive pulmonary disease; CPB* = *cardiopulmonary-bypass; EuroSCORE* = *European System for Cardiac Operative Risk Evaluation; LVEF* = *left ventricular ejection fraction; min* = *minutes*.

### Effect of comorbidities and concomitant medication on pre-operative DPP4 activity

**Figure 2:**
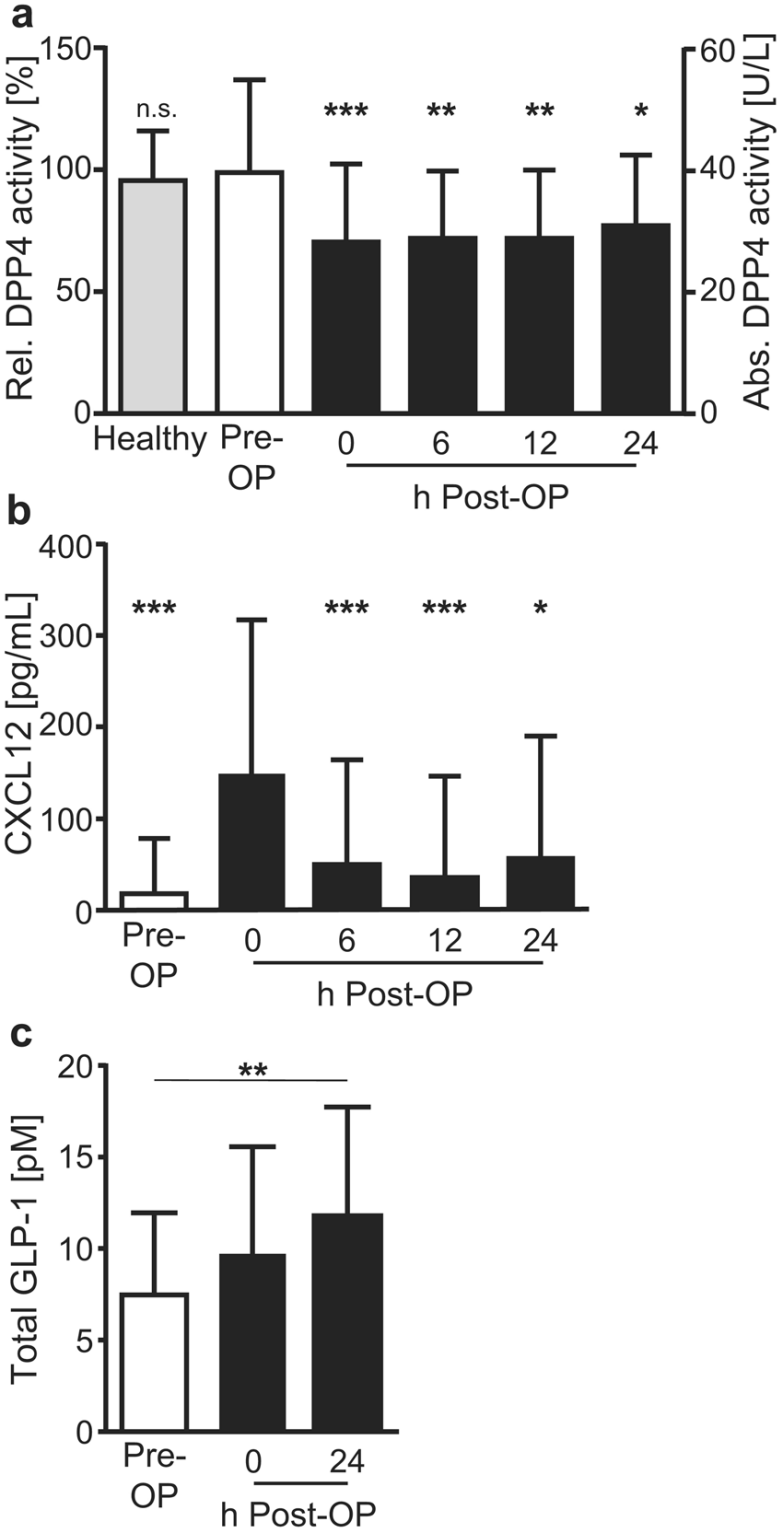
Perioperative analysis of DPP4 activity, CXCL12 and GLP-1. Serum samples were collected before (pre-OP) or at different time points after surgery (0, 6, 12, 24 h post-OP). (**a**) DPP4 activity in serum samples. n = 6 (controls); 29 ≤ n ≤ 44 (patients). Statistical significance is indicated relative to pre-OP values. (**b**) CXCL12 serum levels before and at different time points after surgery. 26 ≤ n ≤ 42. Statistical significance is indicated relative to ‘0 h post-OP’ values. (**c**) Serum levels of total GLP-1 before and at different time points after surgery. n = 9. (**a**–**c**) Shown are means ± SD. One-way ANOVA (Kruskal-Wallis) with Dunn’s post-test. *P < 0.05; **P < 0.01; ***P < 0.001. *n*.*s*. = *not significant*.

Patients scheduled for cardiac surgery showed comparable DPP4 activity prior to surgery when compared to a group of healthy volunteers without any pre-existing disease (male/female = 50%/50%; age 28 ± 4; n = 6) (Fig. 2a; absolute pre-operative DPP4 activity in serum: patients 38.30 (±14.74) U/L vs. healthy controls 37.00 (±7.9), the latter in the range as previously reported^30,31^). Also, patients with T2DM did not show altered DPP4 activity when compared to patients without T2DM (Suppl. Figure 1a). Similarly, body mass index, age and smoking did not significantly affect pre-operative DPP4 activity (Suppl. Figure 1b–d).

### Peri-operative profile of DPP4 activity, CXCL12 and GLP-1 in patients undergoing cardiac surgery

Cardiac surgery induced a significant increase in leukocyte counts in the blood, with a maximal 1.7-fold increase observed directly after surgery (Suppl. Figure 2). Investigating the peri-operative profile of DPP4 activity, we observed a significant decrease with 29% (±25%) immediately after termination of surgery (equaling admission to the ICU) and the DPP4 activity remained significantly lower as compared to pre-operative levels until 24 h after surgery (Fig. 2a). No significant differences were observed in peri-operative DPP4 activity between patients without vs. with T2DM, with a significant reduction in post-operative DPP4 activity observed for both patients groups (6 h/12 h post-OP; Suppl. Figure 1a). Also, no significant association could be detected between either the post-operative DPP4 activity (24 h post-OP, as % of the pre-OP DPP4 activity) or the administration of fluids, blood loss or urine production (Suppl. Figure 3), suggesting that the observed peri-operative decrease in DPP4 activity is not mainly caused by peri-operative fluid management. Simultaneously with an intra-operative decrease in DPP4 activity, we observed an almost 8-fold increase in CXCL12 serum levels after termination of surgery, although serum levels of CXCL12 again returned to baseline 6 h after admission to the ICU (Fig. 2b). Serum levels of total GLP-1 also increased after surgery, reaching a 1.6-fold increase 24 h after operation compared to pre-OP levels (Fig. 2c).

**Figure 3:**
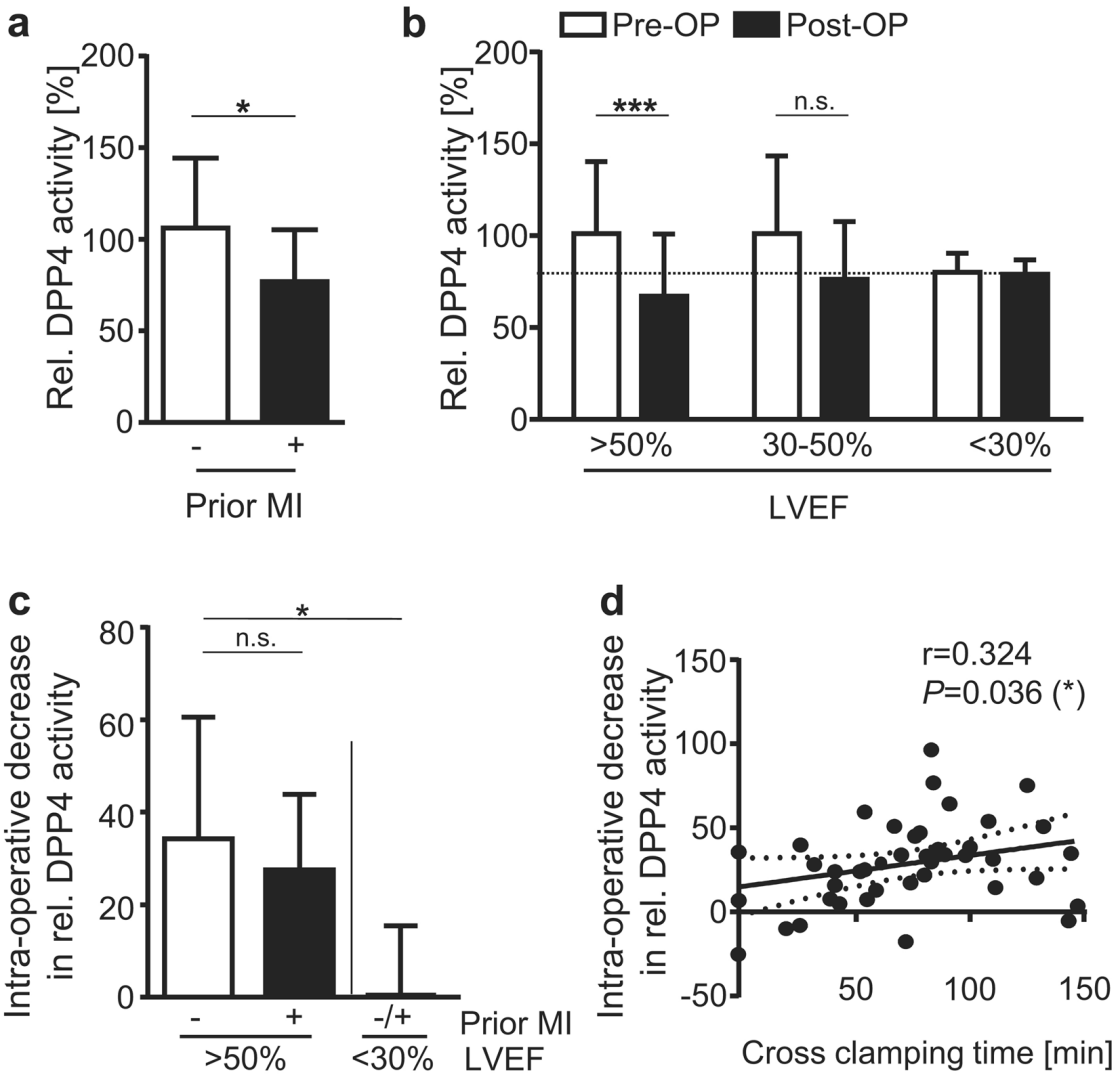
A larger intra-operative decrease in DPP4 activity is observed for patients with better heart function and increased time of aortic cross clamping. (**a**) Relative pre-OP DPP4 activity in patients that did *vs*. did not experience myocardial infarction (MI) within the 90 days prior to cardiac surgery. 12 ≤ n ≤ 32. T-test. (**b**) Relative DPP4 activity in serum of patients with different LVEF, before (pre-OP) and directly after OP (post-OP). 32 ≤ n ≤ 33 (LVEF > 50%); 4 ≤ n ≤ 8 (other). Two-way ANOVA with Sidak’s post-test for comparison of pre- *vs*. post-OP values. (**c,d**) Intra-operative decrease in relative DPP4 activity (pre-OP minus 0 h post-OP): (**c**) in serum of patients according to LVEF and occurrence of a prior MI within the 90 days prior to cardiac surgery. n = 24 (LVEF > 50% no prior MI); 5 ≤ n ≤ 8 (other); one-way ANOVA (Kruskal Wallis) with Dunn’s post-test; (**d**) in function of aortic cross clamping time. a-c, Shown are means ± SD. (**d**) Data are depicted as linear regression (black line) with 95% confidence intervals (dashed lines). r = Spearman correlation coefficient; two-tailed *P*-value. (**a**–**d**) *P < 0.05; **P < 0.01; ***P < 0.001; *n*.*s*. = *not significant*. *LVEF* = *left ventricular ejection fraction; MI* = *myocardial infarction*.

Patients experiencing a myocardial infarction within 90 days prior to the day of surgery exhibited a significant 27% (±11%) lower pre-operative DDP4 activity when compared to patients without recent infarction (Fig. 3a). This was mainly pronounced in patients with conserved left ventricular (LV) function (LV ejection fraction (LVEF) >50%), in whom prior myocardial infarction significantly reduced pre-operative DPP4 activity levels by 29% (±14%) towards the lower level observed in patients with severely reduced LV function (LVEF < 30%) (Suppl. Figure 4). Based on these findings, we compared the intra-operative DPP4 activity profile in patients with preserved vs. reduced left ventricular (LV) function, the latter closely related to ischemic cardiomyopathy. This revealed that a significant intra-operative DPP4 decrease (by 32% ± 24%) was only detectable in patients with preserved LV function (LVEF > 50%). In contrast, DPP4 activity did not change during the course of surgery of patients with severely reduced left ventricular function (LVEF < 30%) (Fig. 3b,c). Further, within the patient group with preserved LV function, no difference in intra-operative DPP4 activity decline was found in patients without vs. with prior myocardial infarction (Fig. 3c). Altogether, these data suggest that the extent of intra-operative DPP4 activity decrease during surgery is dependent on the patients’ pre-operative LV function.

Furthermore, a correlation analysis revealed a larger decrease in intra-operative DPP4 activity for an increased duration of aortic cross clamping, representing the duration of myocardial ischemia during surgery (Fig. 3d). In contrast, no significant correlation could be detected between intra-operative DPP4 activity decrease and overall duration of the surgical procedure, indicating that DDP4 activity is independent from the surgical procedure (Suppl. Figure 5).

In summary, a larger intra-operative decrease in DPP4 activity is observed for patients with preserved vs. severely reduced heart function and with increased time of aortic cross clamping.

### Clinical impact of DPP4 activity on the outcome of patients

**Figure 4:**
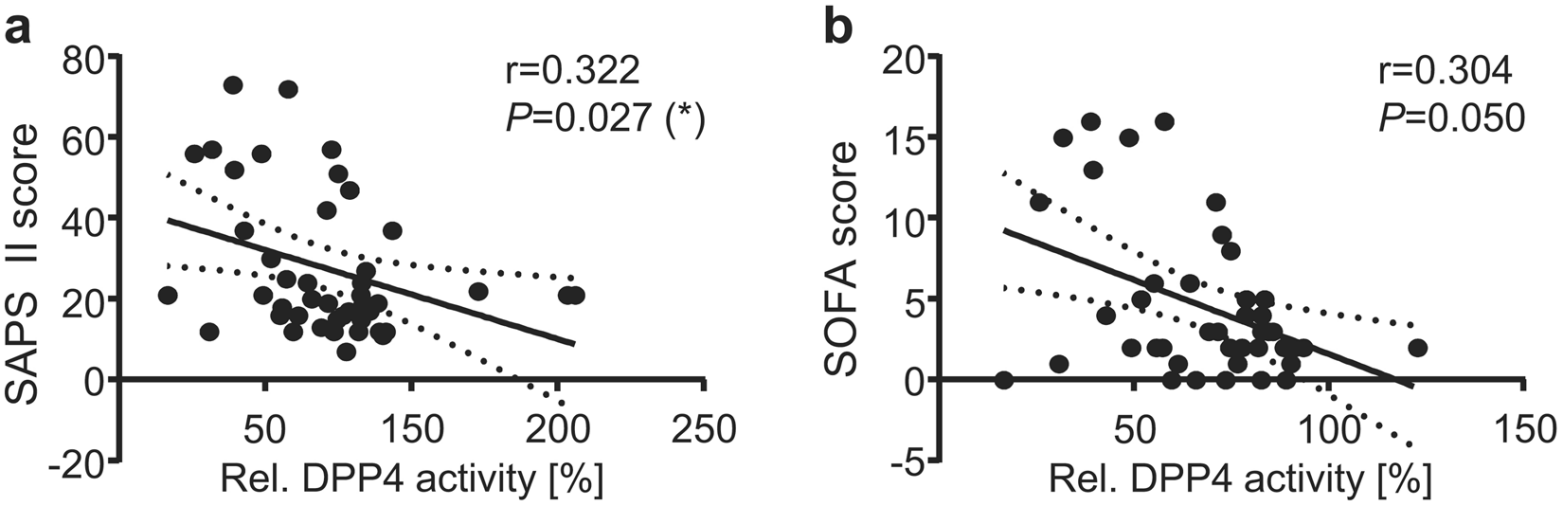
DPP4 activity levels negatively correlate with post-operative morbidity scores. Correlations between relative serum DPP4 activity levels 12 h post-OP and SAPS II score (**a**) or SOFA score (**b**) on the first post-operative day. Data are depicted as linear regression (black line) with 95% confidence intervals (dashed lines). r = Spearman correlation coefficient; two-tailed *P*-value; *P < 0.05.

**Figure 5:**
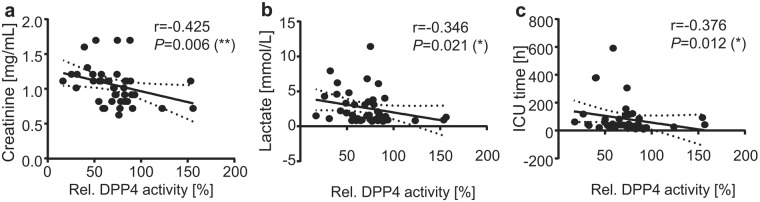
DPP4 activity levels negatively correlate with post-operative organ morbidity and ICU stay. (**a**,**b**) Correlations between relative serum DPP4 activity levels 12 h post-OP and circulating creatinine levels (**a**) and lactate levels (**b**) on the first post-operative day. (**c**) Correlations between relative serum DPP4 activity levels 12 h post-OP and ICU stay. **(a**–**c**) Data are depicted as linear regression (black line) with 95% confidence intervals (dashed lines). r = Spearman correlation coefficient; two-tailed *P*-value; *P < 0.05; **P < 0.01.

We next evaluated the clinical impact of the peri-operative DPP4 activity profile and assessed potential clinical relevant associations of the pre- and post-operative DDP4 activity to the occurrence of organ dysfunctions after surgery. No significant correlation was detected between pre-operative DPP4 activity and post-operative organ injury evaluated by the well-established SAPS II and SOFA score^32^ (Suppl. Table 1). In contrast, DPP4 activity levels measured 12 h after surgery revealed a significant inverse correlation with both the SAPS II and the SOFA score on the first post-operative day (Fig. 4a,b). Furthermore, an inverse correlation was observed between the 12 h post-operative DPP4 activity and post-operative levels of creatinine and lactate as markers for kidney dysfunction, respectively, insufficient oxygen supply and microcirculatory dysfunction (Fig. 5a,b). Also, DPP4 activity after surgery inversely correlated with the patients’ duration of ICU stay (Fig. 5c). Altogether, these data indicate that high post-operative DPP4 activity is associated with a preservation of the patients’ organ function and a more beneficial short-term outcome after cardiac surgery.

### DPP4 oxidation is induced during cardiac surgery and decreases DPP4 activity

**Figure 6:**
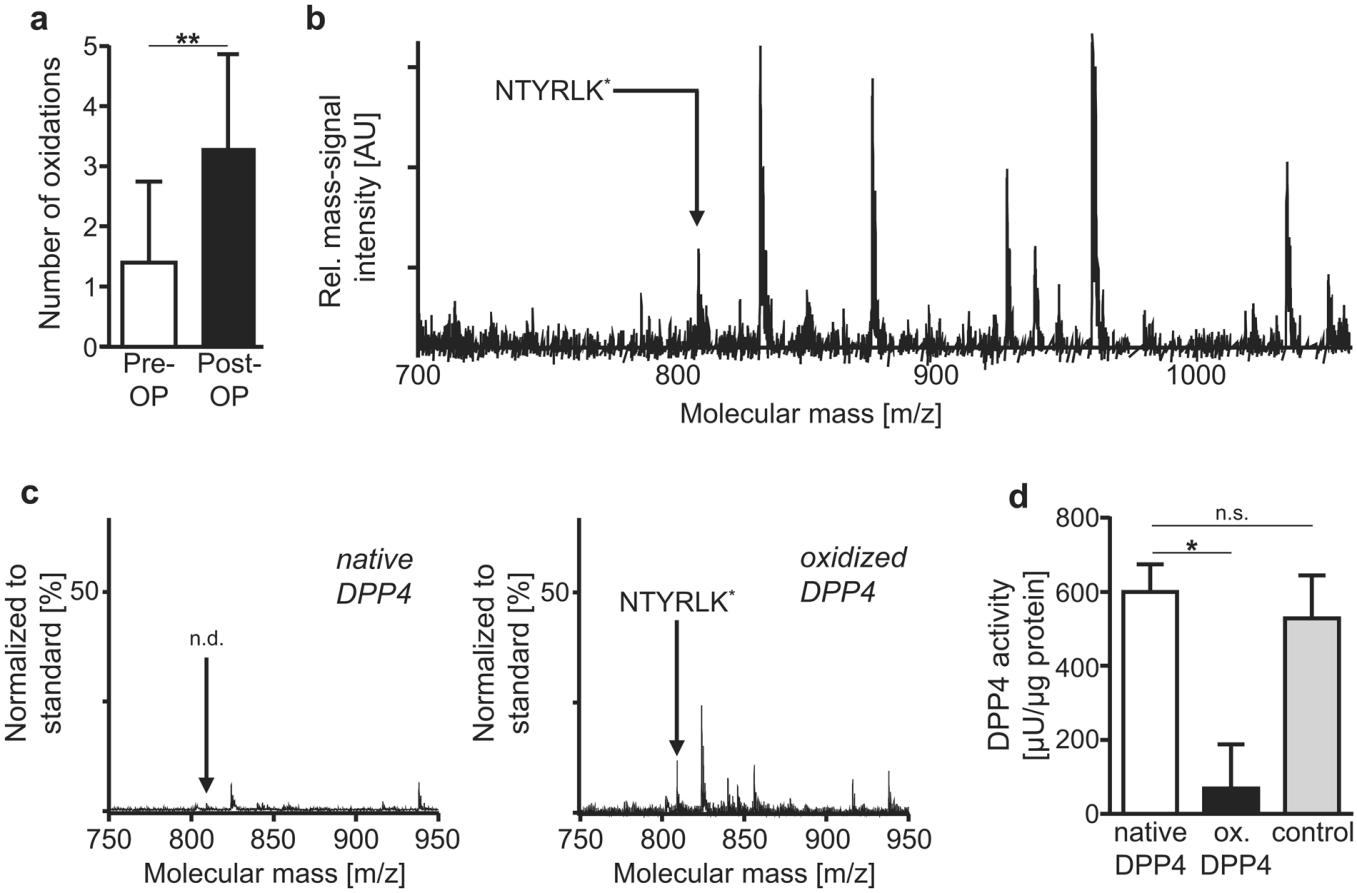
DPP4 oxidation is induced during cardiac surgery and decreases DPP4 activity. (**a**) Quantification of oxidized amino acids of DPP4 peptides from serum of patients before *(Pre-OP)* vs. after *(Post-OP)* cardiac surgery (oxidized amino acids: lysine, histidine, methionine, tryptophane) using MALDI mass-spectrometry (n = 10). Shown are means ± SD. Two-tailed t-test. **P < 0.01. (**b**) Characteristic MALDI-TOF mass-spectrum of trypsinized DDP4 protein isolated from serum of a patient after cardiac surgery. The arrow at 809.2 Da indicates the mass signal of the oxidized DDP4fragment NTYRLK^*^, with K* the oxidized amino acid lysine. (**c**) Characteristic mass fingerprint spectra of trypsinized recombinant human DPP4, before (left) and after (right) *in vitro* oxidation with H_2_O_2_ (representative for 3 experiments). The arrow in the right panel indicates the mass signal of the oxidized DDP4 fragment NTYRLK^*^ at 809.2 Da, which was not detected (n.d.) before oxidation (left panel). Mass signals were normalized to the internal standard (C^13^ Ang II). (**d**) Quantification of the activity of *in vitro* oxidized DPP4 (ox. DPP4) *versus* native recombinant human DPP4. To evaluate potential effects of residual H_2_O_2_ after extensive dialysis on DPP4 activity, a “H_2_O_2_ control sample” lacking DPP4 was dialyzed in the same way, after which DPP4 was added to the same final concentration as the oxidized DPP4 after dialysis *(“control”)*. n = 3. Shown are means ± SD. One-way ANOVA (Kruskal Wallis) with Dunn’s post-test. *P < 0.05; n.s. = not significant.

As myocardial ischemia and ischemia/reperfusion injury induces oxidative stress^33^ and post-translational oxidation can crucially affect protein functions, we examined whether cardiac surgery may affect DPP4 by post-translational oxidation. MALDI mass spectrometric analysis revealed that cardiac surgery significantly increased the oxidation level of serum DPP4 (Fig. 6a), with Fig. 6b displaying a characteristic MALDI-TOF mass-spectrum of trypsinized DDP4 protein isolated from serum of a patient after cardiac surgery. The arrow at 809.2 Da indicates the mass signal of the oxidized DDP4-fragment NTYRLK^*^, with K* the oxidized amino acid lysine.

Further, *in vitro* post-translational oxidation of human recombinant DPP4 with H_2_O_2_ (Fig. 6c) significantly reduced its enzymatic activity (Fig. 6d). To control for potentially adverse effects of residual H_2_O_2_ after extensive dialysis on DPP4 activity, a control sample with H_2_O_2_ but lacking DPP4 was dialyzed in the same way, after which DPP4 was added to the same final concentration as the oxidized DPP4 after dialysis. DPP4 activity measurements demonstrated that potentially residual H_2_O_2_ after extensive dialysis did not affect DPP4 activity (“control” in Fig. 6d).

Combined, these data indicate that DPP4 oxidation is induced during cardiac surgery and significantly decreases DPP4 activity.

## Discussion

**Figure 7:**
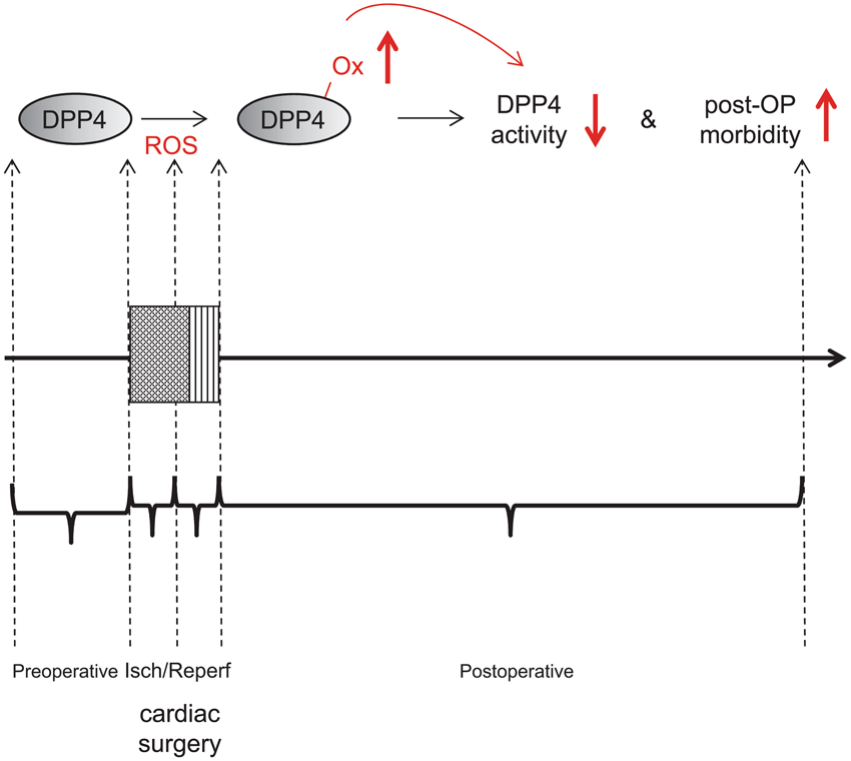
Schematic representation of the main findings of this study. Cardiac surgery increases DPP4 oxidation (*DPP4-ox*) through the production of reactive oxygen species (ROS) and reduces DPP4 activity in the patients’ serum, with low post-operative DPP4 activity levels associated with worse outcome during ICU stay.

Patients undergoing cardiac surgery with CPB are frequently exposed to myocardial I/R injury after termination of cardioplegic-induced myocardial arrest and restoration of the coronary blood flow. This triggers a peri-operative inflammatory response, which further contributes to the development of acute organ dysfunctions during the postoperative course^7,8^. The present study is the first to our knowledge investigating the peri-operative DPP4 activity profile in patients undergoing cardiac surgery with use of CPB and its clinical relevance in relation to post-operative organ dysfunctions. Our data reveal that cardiac surgery enhances DPP4 oxidation and reduces DPP4 activity in serum, with low post-operative DPP4 activity levels being associated with a worse patient outcome during ICU stay (Fig. 7).

Specifically, the post-operative DPP4 activity levels revealed a significant negative correlation with i) the post-operatively assessed SAPS II and SOFA scores, two well-established organ failure scores representing the extent of organ dysfunction^32,34^ and therefore frequently used to asses organ dysfunction throughout ICU stay in the clinical practice. Furthermore, an inverse correlation was observed between post-operative DPP4 activity and ii) post-operative levels of creatinine and lactate as markers for kidney dysfunction, respectively, insufficient oxygen supply and microcirculatory dysfunction, as well as with iii) the patients’ duration of ICU stay. Notably, the organo-protective effects associated with high DDP4 activity were most pronounced when regarding the post-operative kidney functions. This is of particular relevance in cardiac surgery patients, in whom acute kidney injury complicates recovery from cardiac surgery in up to 30% of patients^35^, and which reflects a close interaction of cardio-renal function^36^.

These findings of DPP4 activity in relation to patients’ outcome at the ICU after cardiac surgery are surprising, especially in the context of the preclinical studies in animal models that revealed rather an anti-inflammatory and cardioprotective effect of DPP4 inhibition after myocardial I/R injury^12–19,37^, as well as an antifibrotic effect of DPP4 inhibition in a mouse model of kidney fibrosis^38^. However, our findings demonstrating low DPP4 activity after cardiac surgery to be associated with worse patients’ outcomes during ICU stay focus on the acute clinical situation, which may be different from the chronic clinical outcome. Also, present findings obtained from our observational, clinical study, remain correlative and do not necessarily show a causative relationship. Follow-up large-scale clinical studies are needed to further investigate the clinical impact of DPP4 activity on patients’ mid to long-term outcome after cardiac surgery.

In addition, recent large-scale clinical trials examining the cardiovascular outcome of DPP4 inhibitors demonstrated that these beneficial effects of DPP4 inhibition as observed in animal models could not merely be translated to all clinical relevant patient situations. In these studies, the investigators could not observe a beneficial effect of DPP4 inhibition on the combined risk of cardiovascular death, myocardial infarction and stroke in diabetic patients^22,23,25–27^, nor could in a reproducible way a beneficial renal effect be shown^22^. Thus, combined with our findings, it can be concluded that although DPP4 inhibition clearly confers protective features in animal models, the relation of DPP4 inhibition and also DPP4 activity to patient outcome is not that straightforward in the clinical situation and may vary dependent on the acute vs. chronic clinical situation, as well as on the individual patient cohort. The latter became also obvious in a recent study by Li *et al*. demonstrating low plasma DPP4 activity to be independently associated with left ventricular systolic dysfunction in patients with ST-segment elevation myocardial infarction (STEMI)^39^. These findings are in line with our study reporting low post-operative DPP4 activity to be associated with a worse patient outcome after cardiac surgery, while the myocardial I/R injury following surgery may be in analogy to STEMI patients. However, in contrast to these patient groups, others reported on a correlation of high DPP4 activity with cardiac dysfunction in human heart failure^20,40^ and with subclinical left ventricular dysfunction in patients with T2DM^41^. The differential findings of these studies vs. the study of Li *et al*.^39^ and our study could be explained by the differential patient group examined with different pathophysiology of the underlying disease: as our data show that DPP4 activity is decreased by prior myocardial infarction and a larger intra-operative decrease in DPP4 activity is observed for an increased duration of aortic cross clamping, it is likely that the degree of DPP4 activity decrease after myocardial infarction or cardiac surgery reflects the severity of myocardial ischemia, and that thereby low DPP4 activity levels are associated with worse patients’ short term outcome. In our study this is further underlined by the inverse correlation of post-operative DPP4 activity with i) high levels of lactate as marker for insufficient oxygen supply, which may result from inadequate myocardial function, and ii) given the importance of the cardio-renal link^36^, with high creatinine levels as marker of kidney dysfunction. Yet, we acknowledge that present findings only report on patients‘ short-term outcomes, whereas mid to long-term observations might be more elusive to compare the current findings to those of previous studies on DPP4 activity in relation to clinical outcome after longer observation periods. Although such measurements are undoubtedly important, it has been critically discussed which outcome analysis adequately captures the patients’ perspective focusing on quality of life on longer term after cardiac surgery^42–45^, which has to be considered in future studies.

Whether DPP4 substrates may be involved in a link between low DPP4 activity and worse patient outcome after cardiac surgery, remains currently unclear. Serum levels of CXCL12, an important substrate of DPP4 activity^14,15^, significantly increased shortly after termination of surgery in line with previous observations^46^, but again returned to baseline 6 h after admission to the ICU. Serum levels of GLP-1 also increased after surgery and remained significantly increased also 24 h after operation. Although the role of GLP-1 in patient outcome after cardiac surgery remains subject of investigation, enhanced levels of GLP-1 have been associated with cardioprotective functions of DPP4 inhibition in preliminary studies^12,14^. Thus, the increased GLP-1 levels after surgery observed in our study would argue against a causal link between low DPP4 activity and worse patient outcome after cardiac surgery, although it should also be mentioned that serum GLP-1 levels are not influenced solely by DPP4 but also by inflammation-induced secretion of GLP-1^47,48^.

Further, it is increasingly being debated whether other DPP4 substrates may counteract the overall protective effects of GLP-1, which may explain why cardiovascular outcome trials could not detect a beneficial effect of DPP4 inhibition^12,49^. Of note, several studies have identified specific detrimental effects of DPP4 inhibition that contrast the mainly beneficial actions of DPP4 blockade reported thus far. For example, in a rat model of critical limb ischemia, *DPP4*-deficiency surprisingly reduced angiogenesis, endothelial function and circulating endothelial progenitor cell numbers, suggesting a negative effect of DPP4 inhibition on vascular function and tissue perfusion in this experimental setup^50^. Also, reduced DPP4 levels in endothelial cells upon ischemia, including myocardial infarction, induce a pro-thrombotic status of the endothelium through enhanced tissue factor expression and platelet adhesion^51^, which may have detrimental implications for patients with myocardial ischemia. However, given the explorative nature of this study, we acknowledge that present results should be interpreted cautiously within the limitations of a pilot study. Following studies thus are needed in an adequately designed large scale clinical trial to clarify if specific DPP4 substrates may be involved in the observed association of low post-operative DPP4 activity levels and worse patients’ outcome after cardiac surgery.

It has been reported that in healthy individuals, plasma DPP4 activity is positively correlated with BMI^52^. Also, serum DPP4 levels were shown to correlate with the amount of visceral adipose tissue^53^. However, in our study cohort, no significant correlation was detected between BMI and serum DPP4 activity. This is in accordance with previous findings in STEMI patients, in whom no difference was detected in BMI among different quartiles of plasma DPP4 activity^39,54^. In contrast, as mentioned earlier, patients with a recent myocardial infarction exhibited a significantly reduced pre-operative DDP4 activity when compared to patients without recent infarction. Further, DPP4 activity decreased during cardiac surgery with CPB, if not already decreased by a recent myocardial infarction or severely reduced LVEF. Although this contrasts earlier experimental findings of hypoxia-induced shedding of membrane DPP4 from vascular cells^20,55^, our findings are in line with a clinical study in STEMI patients showing reduced plasma DPP4 activity after myocardial infarction^54^. Also, hypoxia treatment of adipocytes significantly reduced the activity of shedded DPP4^56^, although the underlying mechanisms remained unclear. Post-translational modifications can crucially affect protein functions, and alterations in post-translational modifications have been linked with disease states including myocardial ischemia^57^. For example, it was shown that myocardial ischemia transiently increases O-linked β-*N*-acetylglucosamine attachments (*O*-GlcNAcylation) that provide cardioprotection^58^. In our study, mass spectrometric analyses revealed that cardiac surgery increased the post-translational oxidation of serum DPP4, the latter reducing DPP4 activity as confirmed *in vitro* within the current study. With myocardial ischemia and ischemia/reperfusion injury generating reactive oxygen species^33^, we thus hypothesize that cardiac surgery-induced ischemia reduces DPP4 activity by oxidative stress-induced DPP4 oxidation (Fig. 7).

In conclusion, our study reveals for the first time that DPP4 activity decreases during cardiac surgery dependent on the duration of surgery-induced myocardial ischemia, and that low post-operative DPP4 activity levels are associated with worse patient outcome during ICU stay. As DPP4 oxidation by hydrogen peroxide reduces DPP4 activity, low post-operative DPP4 activity levels likely reflect the severity of myocardial ischemia through the generation of reactive oxygen species. This may explain why in this patient group DPP4 activity has a differential associative relationship with organ dysfunction compared to patients with T2DM and cardiovascular disease. Additional studies are warranted to identify in more detail the underlying mechanisms in combined experimental and clinical studies and to evaluate potential beneficial effects of anti-oxidative treatment in patients undergoing cardiac surgery.

## Methods

### Materials, data and associated protocols are available upon requests

#### Study design and patients

This is a prospective observational study, which was approved by the institutional review board (Ethics committee, RWTH Aachen University) and performed in accordance with the relevant guidelines and regulations. The study was registered at ClinicalTrials.gov (ClinicalTrials.gov identifier: NCT02488876). Between February and July 2013, patients scheduled for elective cardiac surgery with the use of cardiopulmonary bypass (CPB) were consecutively enrolled in the study after obtainment of written informed consent. Exclusion criteria were emergency operations, known or suspected pregnancy, patients’ age less than 18 years, and failure to obtain written informed consent. All experiments are conform to the principles outlined in the Declaration of Helsinki.

#### Management of anesthesia

All included patients received balanced anesthesia according to institutional routine. Induction of anesthesia was performed with propofol (1.5 mg·kg^−1^) and sufentanil (0.5–1 µg·kg^−1^). Muscle relaxation was obtained with rocuronium (1 mg·kg^−1^). Anesthesia was maintained by continuous infusion of sufentanil (1 µg·kg^−1^·h^−1^) and sevoflurane (0.5–1% minimum alveolar concentration (MAC)). The inhalative agent was switched to continuous administration of propofol (1–1.5 mg·kg^−1^) on bypass and continued throughout the whole procedure. Basic fluid substitution was performed with 1 ml·kg^−1^·h^−1^ balanced crystalloid solutions. Additional fluids, vasopressors or inotropic drugs, were administered according to the discretion of the responsible physicians and local standards.

#### Surgical procedure

After midline sternotomy, dissection of the internal mammary artery and harvesting of the venous conduits, heparin was administered (300 IE·kg^−1^) to obtain an activated clotting time of >480 s. The extracorporeal circulation was performed with a non-pulsatile pump flow of 2.2 L·min^−1^·m^−2^, and blood pressure was maintained between 50 and 70 mm Hg. A single antegrade infusion of cold crystalloid cardioplegic solution was used for induction of cardiac arrest (Custodiol^TM^; Köhler Chemie, Alsbach-Hähnlein, Germany) in the aortic root immediately after aortic cross-clamping. After weaning from CPB, heparin was antagonized with protamine in a ratio of 1:1, and aspirin was administered orally starting 8 h postoperatively.

### Data collection

Prior to surgery most relevant demographic and baseline characteristics, such as age, gender, pre-existing diseases, co-medication and the EuroSCORE (European System for Cardiac Operative Risk Evaluation)^59^ for preoperative risk stratification were recorded. Further relevant clinical data with respect to surgery and postoperative outcome were documented. The incidence of organ dysfunction, systemic inflammatory response syndrome, sepsis, severe sepsis and septic shock was recorded according to the ACCP/SCCM consensus conference criteria^60^. The simplified acute physiology score (SAPS II)^32^ and the sequential organ failure assessment (SOFA) score^34^ were evaluated directly at admission to the intensive care unit (ICU) (0 h post-OP) as well as on the first post-operative day (24 h post-OP). Clinical laboratory parameters (creatinine, lactate etc.) were quantified and recorded from the patient data management system. Also, the duration of surgery, aortic cross clamp time and the patients’ duration of ICU stay were documented.

### Blood sample acquisition

In addition to clinical routine measurements, serum samples were drawn after induction of anesthesia, after connection to the CPB, 2 min after opening of the aortic cross-clamp (myocardial reperfusion), at admission to the intensive care unit (ICU) as well as 6 h, 12 h and 24 h after admission to the ICU. All blood samples were immediately centrifuged (900 g, 10 min, room temperature) and the supernatants were transferred to cryotubes. Subsequently, the serum samples were stored at −80 °C until analysis.

### Measurement of DPP4 activity in serum samples

DPP4 activity levels in patient serum samples and healthy control volunteers were measured as relative light units using the luminescent DPPIV-Glo™ protease assay (Promega, Germany) according to the manufacturer’s instructions, using 25 µl of 300 times diluted serum with 25 µl assay reagent. Relative DPP4 activity levels were expressed as % after normalization to relative pre-OP values at study start.

In addition, absolute DPP4 activity in the patient samples was calculated based on the absolute DPP4 activity of the reference sample used for generating the standard row in the luminescent DPPIV-Glo™ protease assay during the measurement of the patient samples. Absolute DPP4 activity of this reference sample was quantified as the rate of cleaving the fluorescent 7-Amino-4-Methyl Coumarin (AMC) from the non-fluorescent substrate H-Gly-Pro-AMC, using a standard curve for free AMC (Sigma) and following the manufacturer’s instructions (Sigma, Technical Bulletin to DPP4 Activity Assay Kit, Catalog Number MAK088). One unit of DPP4 is defined as the amount of enzyme that cleaves 1 µmol of AMC per minute at 37 °C (U/L = µmol/min/L).

### Measurement of GLP-1 activity in serum samples

Total GLP-1 serum levels were quantified in the presence of 19 µM sitagliptin with a chemiluminescent enzyme-linked immunosorbent assay (ELISA) as previously described^61^. In short, we used as primary antibody HYB 147–06, which reacts with the amidated C-terminus of GLP-1(1–36)-amide, GLP-1(7–36)-amide and GLP-1(9–36)-amide, and as detection antibody biotinylated HYB 147–12, which reacts with a mid-molecular epitope present in all GLP-1 forms.

### DPP4 *in vitro* oxidation

DPP4 was oxidized *in vitro* by incubating 0.021 µg/µl recombinant human DPP4 (Sigma Aldrich, Germany) with 100 mM H_2_O_2_ in 10 mM Tris-HCl buffer for 1.5 h at room temperature. After extensive dialysis using the Pur-A-Lyzer^TM^ Maxi Dialysis Kit (Sigma Aldrich, Germany) against PBS to remove H_2_O_2_, DPP4 protein content was measured using the *DC*^TM^ Protein Assay (Bio-Rad, Germany) according to the manufacturer’s instructions. Native DPP4 used as control was treated in the same way but without addition of H_2_O_2_. Also, to evaluate potential effects of residual H_2_O_2_ after extensive dialysis on DPP4 activity, a “H_2_O_2_ control sample” lacking DPP4 was dialyzed in the same way, after which DPP4 was added to the same final concentration as the oxidized DPP4 after dialysis. Then, DPP4 activity was measured using the DPPIV-Glo™ protease assay (Promega, Germany) according to the manufacturer’s instructions, and DPP4 activity was expressed as µU/µg DPP4 protein.

### Quantification of oxidized DPP4 by matrix assisted laser desorption/ionisation time of flight mass spectrometry

The DPP4 protein within the serum of patients before and after cardiac surgery or after *in vitro* oxidation was analyzed for post-translational oxidation by matrix-assisted-laser-desorption/ionisation-time-of-flight-mass spectrometry (MALDI-TOF/TOF) as previously described^62^. Patient serum was supplemented with 4x LDS sample buffer including reducing agent (100 mM DTT), boiled for 10 min at 70 °C, and run on a 15% sodium dodecyl sulphate-polyacrylamide gel electrophoresis gel (SDS-PAGE). Bands were cut out, washed and incubated in the presence of an aqueous ammonium bicarbonate (50 mM) solution and 0.2% w/v trypsin of 24 h at 37 °C. To analyze human recombinant DPP4 after *in vitro* oxidation, the samples were similarly incubated in the presence of an aqueous ammonium bicarbonate (50 mM) solution and 0.2% w/v trypsin of 24 h at 37 °C.

The resulting tryptic peptides were desalted and concentrated by ZipTip_C18_ technology (Millipore, USA) using 0.1% trifluoroacetic acid in water and were eluated by using 80% acetonitrile in water. The eluate was spread onto the (MALDI) target plate (MTP-ground steel 400/384; Bruker-Daltonic, Germany) using α-cyano-4-hydroxycinamic acid (205 mg ml^−1^) as matrix. The subsequent mass spectrometric analyses were carried out using a reflectron-type time-of-flight-mass spectrometer MALDI-TOF/TOF (Ultraflex III; Bruker-Daltonic, Germany). MS/MS fragments were analyzed using Lift-option of the mass-spectrometer. Calibrated and annotated spectra were subjected to the database search Swiss-Prot (http://www.expasy.org/) utilizing the software tool “Bruker Bio-Tool 3.2 and the “Mascot 2.2 search engine” (Matrix Science Ltd, London, UK).

### Statistical analysis

All data were statistically analyzed using a commercially available software package (SPSS 23, IBM; or GraphPad Prism 6, Graphpad Software Inc.). Data are represented as means ± SD. After testing for normality, data were analyzed by Student’s T-test or Mann Whitney Test, as appropriate, for comparison of two groups. For comparison of more than two groups, one-way ANOVA with Tukey’s post-test or a Kruskal-Wallis with Dunn’s post-test was used, as appropriate. A two-way ANOVA was combined with the Sidak’s post-test. Observed differences with *P* < 0.05 were considered to be statistically significant. For correlation studies, linear regression analysis was performed calculating the Pearson or Spearman correlation coefficient, as appropriate, and a corresponding *P*-value. In all cases, a level of *P* < 0.05 was considered statistically significant.

## Electronic supplementary material

Supplementary Information
