# Supplementary material for: Reduced post-operative DPP4 activity associated with worse patient outcome after cardiac surgery
Source: Sci Rep. 2018 Aug 7;8:11820. doi: 10.1038/s41598-018-30235-w (PMC6081383; doi:10.1038/s41598-018-30235-w)
Supplement: Supplementary file 1 — Supplementary Information [file 41598_2018_30235_MOESM1_ESM.pdf]

SUPPLEMENTARY INFORMATION

Reduced post-operative DPP4 activity associated with worse patient outcome after cardiac surgery

Short title: DPP4 activity in cardiac surgery

Heidi Noels[1], Wendy Theelen[1], Marieke Sternkopf[1], Vera Jankowski[1], Julia Moellmann[2], Sandra Kraemer[3], Michael Lehrke[2], Nikolaus Marx[2], Lukas Martin[4], Gernot Marx[4], Joachim Jankowski[1,5], Andreas Goetzenich[3], Christian Stoppe[4].

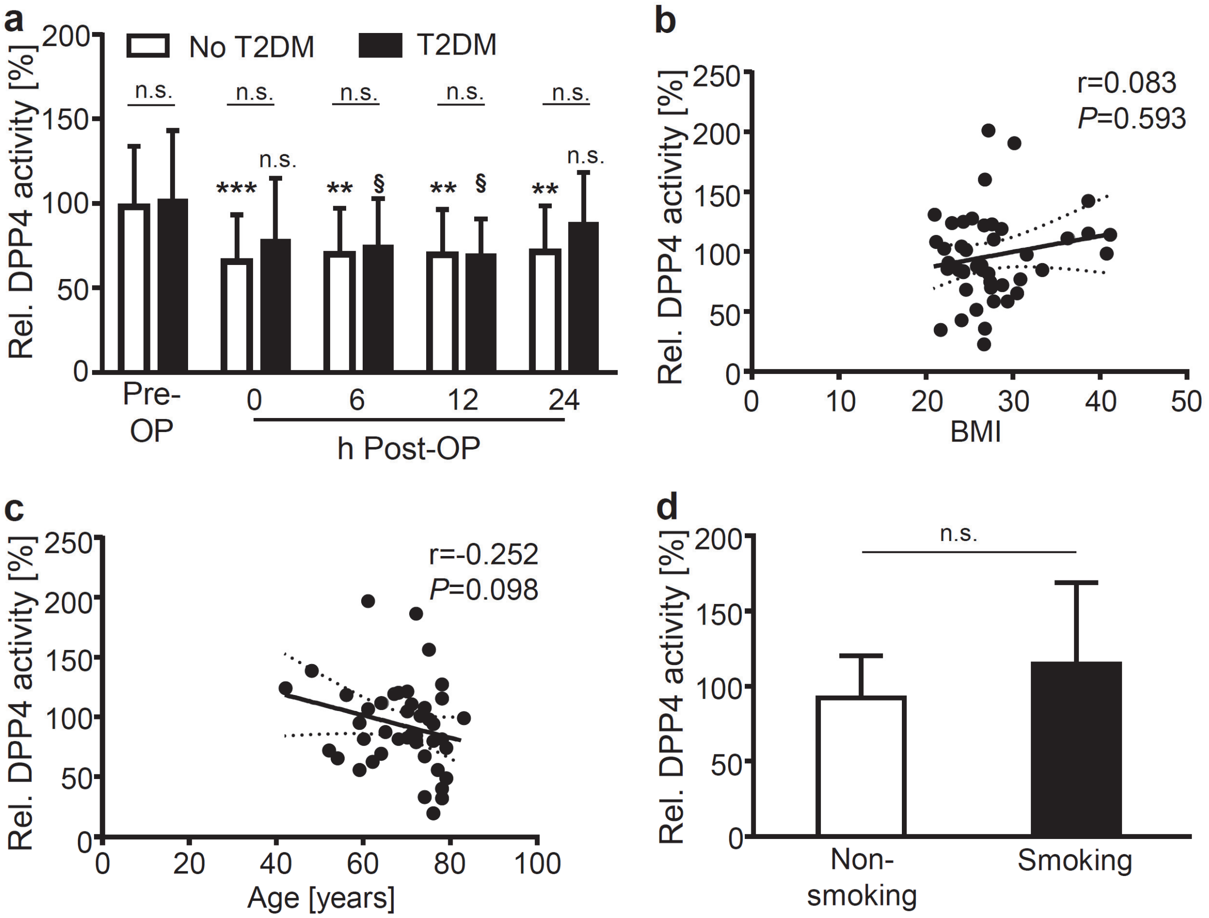

Suppl. Figure 1. Effect of biometric and clinical parameters on pre-operative DPP4 activity. Pre-operative serum DPP4 activity levels were quantified in patients scheduled for cardiac OP. **a,** Relative DPP4 activity levels in serum of patients without *vs.* with T2DM. no T2DM: n=18 (24h post-OP) and 26≤n≤28 (other time points); with T2DM: n=12 (24h post-OP) and 14≤n≤17 (other time points). Two-way ANOVA with Holm-Sidak's post-test to compare i) within each patient group DPP4 activity relative to pre-OP values of the respective patient group (**/*** for patients without diabetes; § for patients without diabetes); and ii) for comparing patients with vs. without T2DM at every time point (n.s. = not significant). **b-c,** Correlation between relative DPP4 activity and BMI (b) or age (c). Data are depicted as linear regression (black line) with 95% confidence intervals (dashed lines). r = Spearman correlation coefficient; two-tailed *P*-value. **d,** DPP4 activity levels in non-smoking *vs.* smoking patients. 12≤n≤32. Unpaired two-tailed t-test. a**, d,** Shown are means ± SD.

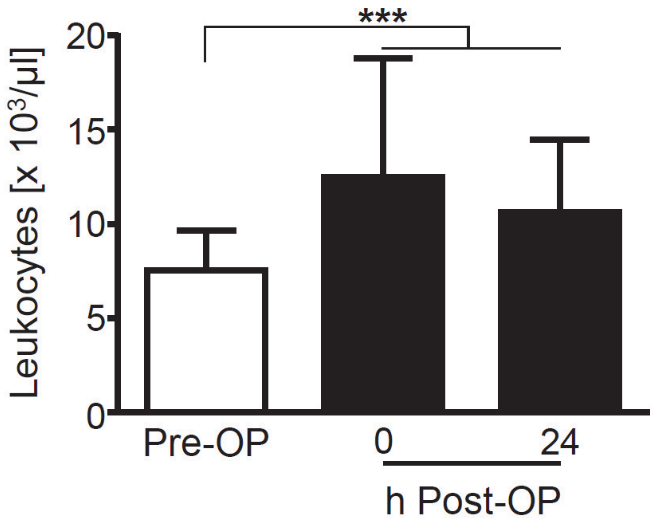

Suppl. Figure 2. Cardiac operation induces a significant increase in circulating leukocyte counts. Leukocytes were quantified in blood before (pre-OP) and at different time points after OP (post-OP), as indicated. Shown are means ± SD. 43≤n≤44. One-way ANOVA (Kruskal Wallis) with Dunn's post-test. ****P*<0.001.

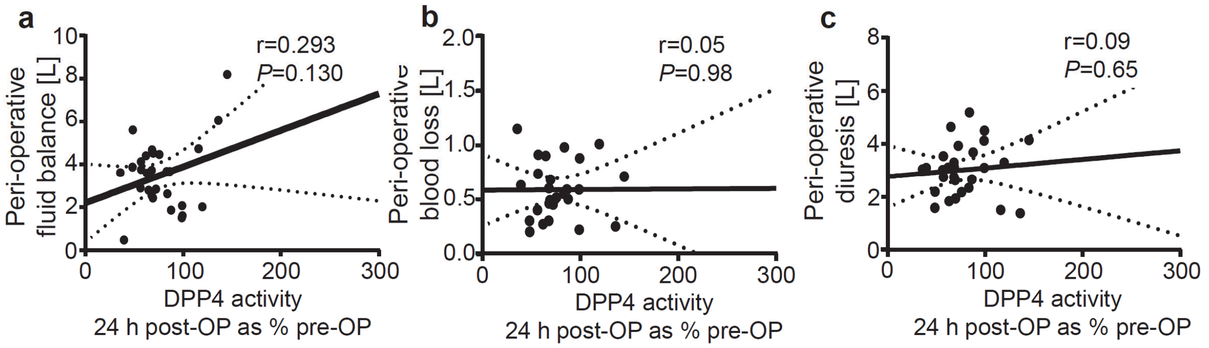

Suppl. Figure 3. Effect of peri-operative fluid administration on DPP4 activity. a-c, Correlation between 24 h post-OP activity as % pre-OP and fluid balance (a), blood loss (b) or peri-operative diuresis (c). Data are depicted as linear regression (black line) with 95% confidence intervals (dashed lines). r = Pearson correlation coefficient; two-tailed *P*-value.

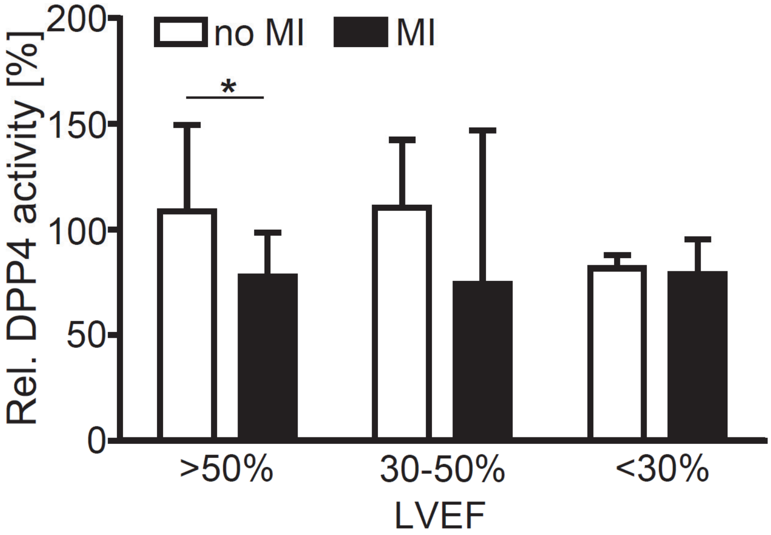

Suppl. Figure 4. Effect of myocardial infarction and cardiac function on DPP4 activity. Relative DPP4 activity levels in patients according to LVEF and occurrence of a prior MI within the 90 days prior to cardiac surgery. 8≤n≤25 (LVEF>50%); 2≤n≤6 (others). Mann-Whitney test; *P<0.05. Depicted are means ± SD.

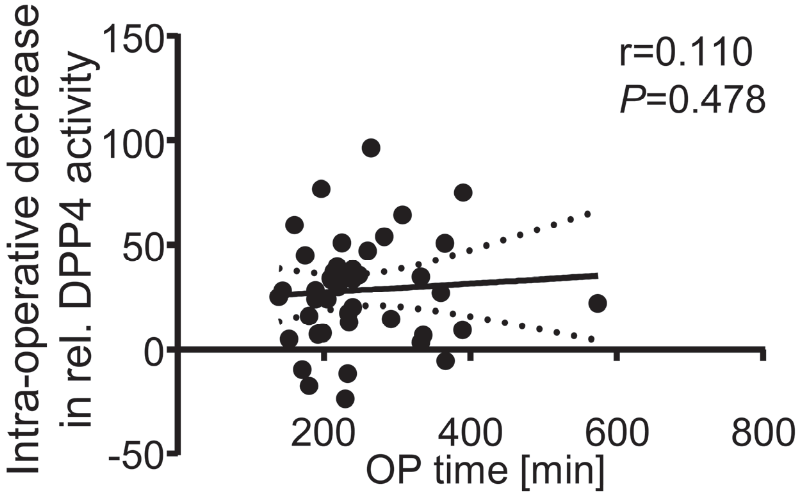

Suppl. Figure 5. No correlation between the intra-operative DPP4 activity decrease and total operation time. Correlation between the intra-operative decrease in relative DPP4 activity (pre-OP minus 0 h post-OP) and the overall duration of the surgery. Data are depicted as linear regression (black line) with 95% confidence intervals (dashed lines). r = Spearman correlation coefficient; two-tailed *P*-value.

Suppl. Table. Correlations between the pre-operative levels of relative DPP4 activity (%) and the SAPS II or SOFA score directly after surgery (0 h post-OP) or 24 h post-OP. *r* = Pearson or Spearman correlation coefficient, as appropriate; two-tailed *P*-value.

Correlations pre-operative DPP4 activity levels *versus x*	*r*	*P*-value
SAPSII (0 h post-OP)	-0.225	0.141
SAPSII (24 h post-OP)	-0.128	0.407
SOFA (0 h post-OP)	-0.108	0.485
SOFA (24 h post-OP)	-0.223	0.146
